# Monitoring the impacts of crop residue cover on agricultural productivity and soil chemical and physical characteristics

**DOI:** 10.1038/s41598-023-42367-9

**Published:** 2023-09-12

**Authors:** Mohammad Kazemi Garajeh, Keyvan Hassangholizadeh, Amir Reza Bakhshi Lomer, Amin Ranjbari, Ladan Ebadi, Mostafa Sadeghnejad

**Affiliations:** 1 Department of Civil, Constructional and Environmental Engineering, Sapienza University of Rome, 00138 Rome, Italy; 2 Department of Remote Sensing and GIS, University of Tabriz, Tabriz, Iran; 3 Department of Geography, Birkbeck, University of London, London, WC1E 7HX UK; 4 Department of Surveying Engineering, Faculty of Engineering, Golestan University, Aliabad Katoul, Iran; 5 Department of Geography and Geospatial Sciences, Kansas State University, 920 N17th Street, Manhattan, KS USA

**Keywords:** Ecology, Environmental sciences, Planetary science

## Abstract

To the best of our knowledge, the impacts of crop residue cover (CRC) on agricultural productivity and soil fertility have not been studied by previous researchers. In this regard, this study aims to apply an integrated approach of remote sensing and geospatial analysis to detect CRC and monitor the effects of CRC on agricultural productivity, as well as soil chemical and physical characteristics. To achieve this, a series of Landsat images and 275 ground control points (GCPs) collected from the study areas for the years 2013, 2015, and 2021 were used. A convolutional neural network (CNN), a class of artificial neural network has commonly applied to analyze visual imagery, was employed in this study for CRC detection in two classes (Not-CRC and CRC) for the years 2013, 2015, and 2021. To assess the effects of CRC, the Normalized Difference Vegetation Index (NDVI) was applied to Landsat image series for the years 2015 (22 images), 2019 (20 images), and 2022 (23 images). Furthermore, this study evaluates the impacts of CRC on soil fertility based on collected field observation data. The results show a high performance (Accuracy of > 0.95) of the CNN for CRC detection and mapping. The findings also reveal positive effects of CRC on agricultural productivity, indicating an increase in vegetation density by about 0.1909 and 0.1377 for study areas 1 and 2, respectively, from 2015 to 2022. The results also indicate an increase in soil chemical and physical characteristics, including EC, PH, Na, Mg, HCO_3_, K, silt, sand, and clay from 2015 to 2022, based on physical examination. In general, the findings underscore that the value of an integrated approach of remote sensing and geospatial analysis for detecting CRC and monitoring its impacts on agricultural productivity and soil fertility. This research can offer valuable insight to researchers and decision-makers in the field of soil science, land management and agriculture.

## Introduction

Crop residue cover (CRC) refers to the deceased crop biomass that remains in fields after crop harvesting^1,2^. CRC is critical for preventing soil erosion and enhancing soil fertility due to its rich nitrogen, potassium, and phosphorus contents^3,4^. CRC has a significant impact on agriculture production and can influence biological production and biodiversity by increasing vegetation density and soil fertility^5,6^. It can also enhance the speed of enzymatic activities, decrease water consumption, lower soil temperature, and reduce air pollution and harmful gas emissions^7–9^. In summary, CRC can contribute to the sustainability of agriculture^10,11^.

Having accurate spatially and temporally resolved information about field-scale CRC and its impacts on vegetation and soil fertility will assist decision-makers in assessing the effectiveness of government conservation programs and voluntary ecosystem service markets. This information will also facilitate the quantification of cropland biogeochemical processes through agro-ecosystem modeling^12,13^. The most conventional method for obtaining information about CRC and agricultural production is through field-based data collection, which involves farmers participating in commercial and governmental programs and annual residue surveys^14–16^. These techniques, however, are time-consuming and labor-intensive. Furthermore, several factors, including the personal judgment of surveyors, the high cost of field surveys, and farmers’ concerns regarding data privacy, have affected the quality and accuracy of the data^17,18^. Additionally, field-based approaches cannot provide timely information on CRC and its impacts over a wide region^19^. Therefore, it is necessary to employ a timely, cost-effective, and accurate approach to map and monitor CRC and its effects on agricultural production.

Remote sensing provides multi-spectral and hyper-spectral datasets for mapping CRC and monitoring vegetation density across large areas^20^. Within the realm of CRC mapping, the primary challenge lies in distinguishing CRC from bare soil^21,22^. This issue has been addressed by identifying specific bands near 2100 nm, leading to the development of several indexes, such as the Cellulose Absorption Index (CAI)^23^, Lignin Cellulose Absorption (LCA)^24^, Shortwave Infrared Normalized Difference Residue Index (SINDRI), Normalized Difference Index (NDI), and Dead Fuel Index (DFI)^25–27^. These indexes, on the other hand, are susceptible to atmosphere and ground features since they are based on spectral reflectance^28,29^. Additionally, the spectral reflectance for CRC indexes can be influenced by the type and shape of crop residues^30^. Many CRC indexes also exhibit reduced sensitivity in areas densely covered by CRC and can saturate to various extents^31^. Soil Adjusted Corn Residue Index (SACRI) and Modified Soil Adjusted Corn Residue Index (MSACRI), for instance, are both sensitive to CRC and become saturated at CRC values up to 0.25^32^. Therefore, learning-based approaches, such as machine and deep learning have been employed to retrieve bio-geophysical variables due to their computational efficiency^32–39^. These approaches excel in capturing the non-linear relationships between input variables and desired outputs^40^. According to the literature review, few studies have investigated the efficiency of deep learning convolutional neural networks for CRC monitoring and mapping. However, they have applied for landuse/cover mapping^41,42^ and crop type identification^43^.

Remote sensing also provides various spectral indexes for monitoring and mapping the structure (i.e., density and complexity) and distribution of green areas^44^. The Normalized Difference Vegetation Index (NDVI) is one of the vegetation indexes that has frequently used to assess the long-term trends of vegetation on a large scale. NDVI proves to be an effective method for monitoring plant productivity over time^45^, investigating the relationship between productivity and biodiversity, and determining vegetation composition and landscape structure^46,47^.

According to the literature, few studies have explored the effects of CRC on agricultural productivity and soil chemical and physical characteristics. Previous researchers have predominantly applied various indexes (e.g., CAI) and machine learning techniques (e.g., Random Forest) for CRC detection and mapping^48,49^. The present research, however, intends to accomplish three primary objectives: (1) map and monitor the effects of CRC on agricultural productivity using NDVI from 2015 to 2022, (2) model and monitor the impacts of CRC on soil chemical and physical characteristics from 2015 to 2022, and (3) examine the efficiency of a convolutional neural network (CNN) in detecting and mapping CRC over the study areas. Landsat series images hold substantial potential for monitoring long-term changes in various features^50,51^. In this regard, Landsat series images used to detect CRC and evaluate its impacts on agricultural productivity and soil fertility in Maragheh and Heris, East Azerbaijan Province, Iran, spanning from 2013 to 2022.

## Materials and methodology

### Materials

To evaluate the impacts of CRC on agricultural productivity and soil chemical and physical characteristics, Landsat images with a spatial resolution of 30 m were utilized for the years 2013/09/28, 2015/08/17, 2019, 2021/08/17, and 2022 (Table 1). A total of 275 ground control points (GCPs) were collected from the study areas using the global positioning system (GPS) and Google Earth, which were then used to construct CNN models. Of these GCPs, 70% were allocated for training the CRC models, while the remaining 30% were employed to validate the accuracy of the CNNs.

**Table 1:** More details regarding the employed Landsat images to monitor the effects of CRC on agricultural productivity and soil chemical and physical characteristics.

Study areas	CRC			NDVI		
1	2013/09/28	2015/08/17	2021/08/17	2015/05/13	2019/05/24	2022/05/23
2	2013/09/28	2015/08/17	2021/08/17	2015/05/13	2019/05/24	2022/05/23

Various variables, including precipitation, soil temperature, and soil moisture, were generated from the Moderate Resolution Imaging Spectroradiometer (MODIS) and employed in this study to properly monitor the impacts of CRC on agricultural productivity and soil chemical and physical characteristics. Monthly and annual data of precipitation rate data were obtained from the General Department of Meteorology of East Azarbaijan Province (www.wamo.ir). We also accessed monthly and annual soil temperature and soil moisture data from https://giovanni.gsfc.nasa.gov.

### Soil sample points collection and laboratory measurement

To assess the effects of CRC on soil chemical and physical characteristics, soil samples were collected from the study areas at the end of June, prior to any plowing of agricultural land, ensuring the pristine condition of the topsoil texture. A total of 74 and 62 GCPs were collected from study areas 1 and 2, respectively. These GCPs were selected based on real field observations, existing digital soil type information, and landuse/cover maps spanning from 2015 to 2022. At each sampling point, four topsoil samples were collected and combined using a soil drill. To record the geographical position of soil sampling points, a portable GPS (UniStrong G120 with a positioning accuracy of 0.5 m) was used. Following collection, all soil samples were properly sealed, labelled, and moved to the laboratory for the measurement of key physicochemical soil attributes. To facilitate accurate analysis, soil samples were fully air-dried and sieved through a 2 mm sieve to eliminate extraneous materials. Using a digital multi-parameter measuring apparatus (Multi 3420 Set B, WTWGmbH, Germany) at a room temperature of 25 °C, soil electrical conductivity and soil water ratio were determined based on the leachate prepared at a soil-to-water ration of 1:2.5.

## Methodology

**Figure 1:**
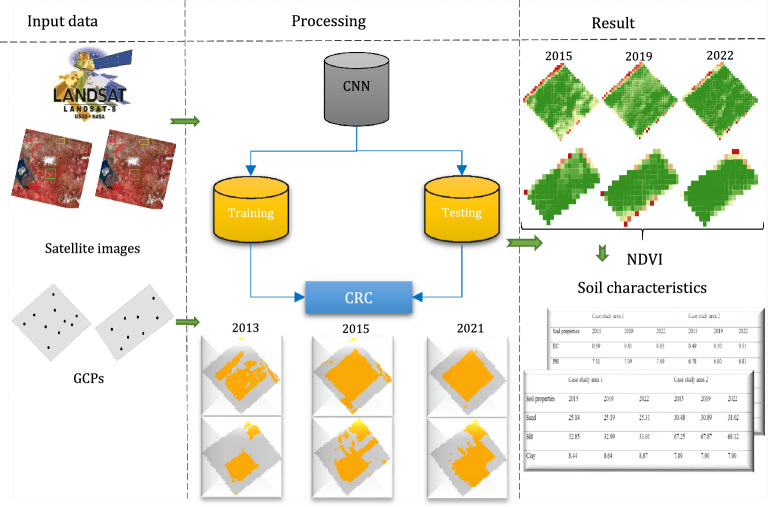
An overview of the employed methodology to evaluate the effects of CRC on agricultural productivity and soil fertility.

This study applied an integrated approach of remote sensing and deep learning data-driven for assessing the impacts of CRC on agricultural productivity and soil chemical and physical characteristics. The methodology consisted of several phase. In the first phase, Convolutional Neural Network (CNN) was utilized to detect all instances of CRC within the study areas. Moving to the second phase, a series of Landsat 8 images were employed to estimate the Normalized Difference Vegetation Index (NDVI), which served as a means to monitor the density of vegetation in the study areas between 2015 and 2022. Additionally, changes in soil chemical and physical characteristics across the study areas from 2015 to 2022 were assessed. Finally, various quantitative approaches were applied to validate the accuracy of the obtained results. Figure 1 reveals an overview of the methodology employed in this study for assessing the effects of CRC on agricultural productivity and soil chemical and physical characteristics.

### Convolutional neural network for CRC mapping

CNN is a neural network architecture inspired by neuroscience findings^52,53^. It has been widely applied in the field of remote sensing for modeling and mapping various Earth features, including landforms^54^, forest fires^55^, soil salinity distribution^56^, landslide^57^, landuse/cover^41^ and more. It is made of layers of artificial neurons called nodes. Each node calculates a weighted sum of inputs and returns an activation map^53,58^. Each node in a layer is defined by its weight values^59^. In the context of images, when a layer receives input, such as an image, it extracts visual properties. Initially, the CNN finds edges in the image. This partial definition of the image is then passed to the next layer, which begins to identify features like corners and color groups. This refined image definition is further processed in subsequent layers until the network makes prediction^60,61^. This research utilized GCPs in conjunction with Landsat 8 imagery, featuring a 30-m spatial resolution, to facilitate the training of CRC models. A comprehensive set of 275 GCPs was used for the model training process. Subsequently, all generated CRC models were categorized into two classes: Not-CRC and CRC. In the realm of input images, elevating the model's input resolution yields enhanced performance. The rationale behind this phenomenon is that the inclusion of finer details (specifically, information) results in improved overall performance. The underlying hypothesis posits that the augmentation of performance with increased image size is solely attributed to the infusion of more intricate details^62^. Table 2 represents further details regarding the applied parameters in CNN.

**Table 2:** Characteristics of employed CNN models for detecting and mapping CRC.

Year			Activation function	Loss function	Optimizer
2013	Case study areas 1 and 2		ReLu	Cross-Entropy	ADAM
2015			ReLu	Cross-Entropy	ADAM
2021			ReLu	Cross-Entropy	ADAM

In CNN, like in other neural networks, there are three layers, namely an input layer, a hidden layer (can be more than one), and an output layer^63^. An input layer is comprised of a $m\times n$ matrix with a respective feature value at each node. A convolutional layer that is immediately adjacent to the input layer is sometimes referred to as a feature extractor. The reason for this is that it is used to extract the features of an image. A back-propagation algorithm optimizes several convolutional kernels in the convolutional layer^64,65^. The output of the convolutional layer will be the input for the next layer^66^. A convolutional layer consists of a pooling layer, multiple weights, and an activation function^67^. Max-pooling is similar to the convolutional layer but instead of taking a dot product between the input and the kernel, it takes the max of the region from the input overlapped by the kernel. In this study, a maximum operator was used to downsample the feature maps in the encoder. Maximum pooling is required to split the feature maps into several rectangular regions for generating maximum values in each layer^68^.

The fully connected layer is used to reduce the loss function and subsequently outputs the classification result^69^. Equations (1)–(3) are defined convolutional manipulation and max pooling, respectively.

1 $$C_{j} = \mathop \sum \limits_{i}^{N} f\left( {w_{j}^{*} v_{i} + b_{j} } \right), j = 1, 2, \ldots , k$$

2 $$f\left( x \right) = \tanh \left( x \right) = \frac{{e^{x} - e^{ - x} }}{{e^{x} + e^{ - x} }}$$

where $f$ denotes the non-linear function, $*$ represents the represented convolutional operator, $k$ denotes convolutional kernels, $w_{j}$ represents the weights, and $b_{j}$ denotes the bias.

3 $$a_{j} = {}_{N \times 1}^{max} \left( {a_{j}^{n \times 1} \mu \left( {n, 1} \right)} \right)$$

where $\mu \left( {n, 1} \right)$ is the window function to patch the earlier layer and $a_{j}$ is the maximum in the patch.

In the next step, a weighted sum was calculated by adding up the convolutional layers. This sum was then passed through an activation function to generate output. The Rectified Linear Unit (ReLu) function employed in this study to construct the CRC model. Equation (4) is defined the ReLu^70^.

4 $$f\left( x \right) = \left\{ {\begin{array}{*{20}c} {0, x < 0} \\ {x, x \ge 0} \\ \end{array} } \right.$$

Finally, all parameters in the CNN were optimized using the back-propagation algorithm. The parameter optimization is applied to reduce the loss function value, which is expressed by Eq. (5). The results obtained from the CRC-CNNs were categorized into two classes: not-CRC and CRC (See Fig. 4).

5 $$L\left( {y, \hat{y}} \right) = - \frac{1}{N}\mathop \sum \limits_{i = 1}^{N} \left( {y_{i} \log \left( {\hat{y}_{i} } \right) + \left( {1 - y_{i} } \right)\log \left( {1 - \hat{y}_{i} } \right)} \right)$$

where $N$ is the total number of the data samples, $y_{i}$ is the actual result of sample $i$ (0 or 1), $\hat{y}_{i}$ is the predicted likelihood of sample $i$ having the output 1, and $y$ and $y_{i}$ are the vectors of real outputs and predicted probabilities.

### Normalized difference vegetation (NDVI) for extracting vegetation density

In numerous studies, satellite-derived vegetation indexes are frequently employed to monitor vegetation status, with the Normalized Difference Vegetation Index (NDVI) being a commonly utilized choice. NDVI serves to gauge the spatial distribution and relative abundance of vegetation^71–73^. These remote sensing-based spectral indexes utilize reflectance measurements gathered from satellites and aircraft to evaluate vegetation status^71^. NDVI is commonly used to measure vegetation cover and is closely associated with chlorophyll content, energy absorption, and photosynthetic capacity^74^. This study applied the NDVI indicator for assessing the impacts of CRC on agricultural productivity from 2015 to 2022, as defined by Eq. (6). The NDVI uses the Red (620–670 nm) and Near Infra-Red (NIR) (841–876 nm) spectrums to estimate vegetation density.

6 $$NDVI = \frac{{\left( {P_{NIR} - P_{Red} } \right)}}{{\left( {P_{NIR} + P_{Red} } \right)}}$$

where $P_{NIR}$ and $P_{Red}$ are top of atmosphere reflectance (TOA) in the NIR and Red bands, respectively. NDVI values range from − 1 to + 1. Since chlorophyll absorbs light, the red spectrum reflection is always lower than the NIR band spectrum reflectance for green vegetation. NDVI values in vegetation regions cannot be less than 0 and below or equal to 0.1 represent water bodies or bare ground due to low reflectance recorded in NIR. The values 0.2–0.5 represent sparse vegetation, while values near 1 represent dense vegetation. Thus this study employed a series of Landsat 8 images (A total number of 65 images) from 2015 to 2022, which were individually converted to NDVI using Eq. (6) for each year.

### Accuracy assessment

Accuracy assessment is a crucial step in the domain of image classification^75^. In this regard, a set of quantitative approaches, including Intersection Over Union (IOU) values, Recall (RC), Precision (PC), Specificity (SP), F-measure (FM), Accuracy (ACC), and Kappa (KP) applied to assess the accuracy of the classification. Equations (7)–(11) describe the employed approaches. The results of the CNN for detecting and monitoring CRC changes also are presented in Table 3. According to the information presented in Table 3, the CNN performs well with the ACC of > 0.95 for CRC mapping.

7 $$IOU = \frac{AO \cap EO}{{AO \cup EO}} = \frac{TP}{{TP + FP + FN}}$$

8 $$Recall = \frac{TP}{{TP + FN}}$$

9 $$Specificity = \frac{TN}{{TN + FP}}$$

10 $$Accuracy = \frac{TP + TN}{{TP + TN + FN + FP}}$$

11 $$Kappa = \frac{{TP + TN - TP_{expected} - TN_{expected} }}{{TP + TN + FN + FP - TP_{expected} - TN_{expected} }}$$

where $AO$ is actual output; $EO$ is on behalf of expected result; $TP$, $FP$, $FN$, and $TN$ are true positive, false positive, false negative, and true negative, respectively.

**Table 3:** The results of CNN for CRC detecting and mapping.

Stud area	Years	IOU	RC	PC	KP	ACC
1	2013	0.8699	0.8845	0.9641	0.9154	0.9685
	2015	0.8700	0.8701	0.9581	0.9014	0.9780
	2021	0.8612	0.8835	0.9600	0.9123	0.9614
2	2013	0.8534	0.8778	0.9514	0.9124	0.9612
	2015	0.8614	0.8701	0.9435	0.99012	0.9564
	2021	0.8542	0.8714	0.9524	0.9089	0.9587

## Study area

### Case study area 1

**Figure 2:**
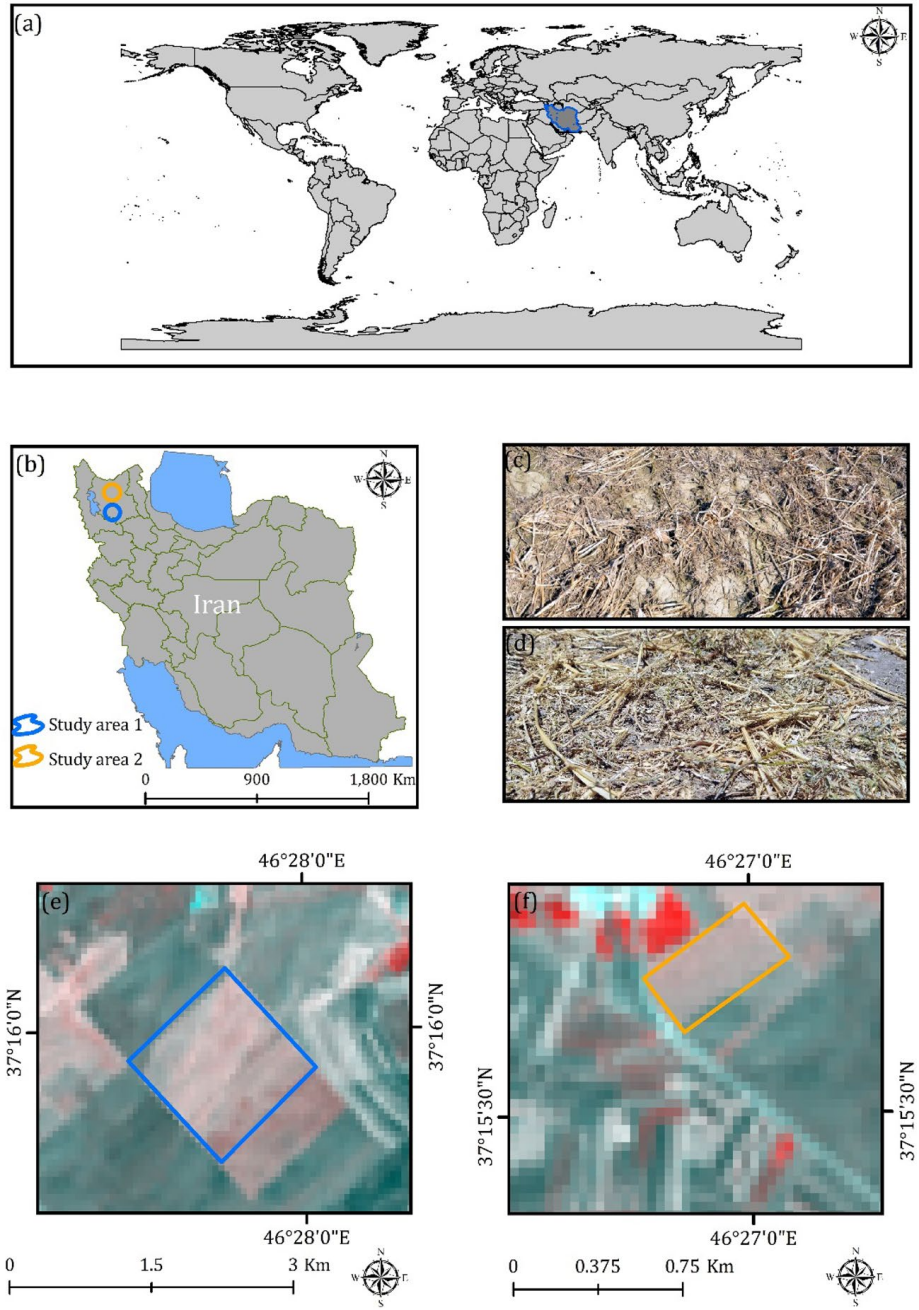
Location of study areas, generated in the ArcGIS 10.6 software (www. esri.com): (**a**) in the world, (**b**) in the north-west of Iran, (**c**) and (**d**) examples of observed CRC over the study areas, (**e**) location of study area in Maragheh city and (**f**) location of study area in Heris city.

The case study area is located at 46° 27ʹ 21ʺ E, 37° 15ʹ 44ʺ N in Maragheh, East Azerbaijan Province, Iran (Fig. 2a, b and e). Clay loam is the main soil texture in the study area. Wheat and pea are the major crops, sometimes in rotation with forage crops. Figure 2c reveals the observed CRC in the case study area 1. Since tillage and planting were mostly conducted under conservation tillage and reduced tillage procedures, different percentages of CRC were readily available. Based on our observation, this area has a regular rotation of products. This convinces us to focus on this area for assessing the impacts of CRC on agricultural productivity and soil chemical and physical characteristics. The annual precipitation rate and temperature are 330 ml and 12.5 °C, respectively for the study area^76,77^.

### Case study area 2

Figure 2a, b and f shows the case study area 2, located in Heris, East Azerbaijan Province, Iran. Wheat is the predominant crop grown in this area. Figure 2d reveals the observed CRC over the case study area 2. From a soil perspective, clay is the dominant soil type in this region. Additionally, this area is characterized as a semi-arid region with hot summers and cold winters. The annual precipitation rate and temperature for this study area are approximately 315 mm and 8.5 °C, respectively.

## Results

**Figure 3:**
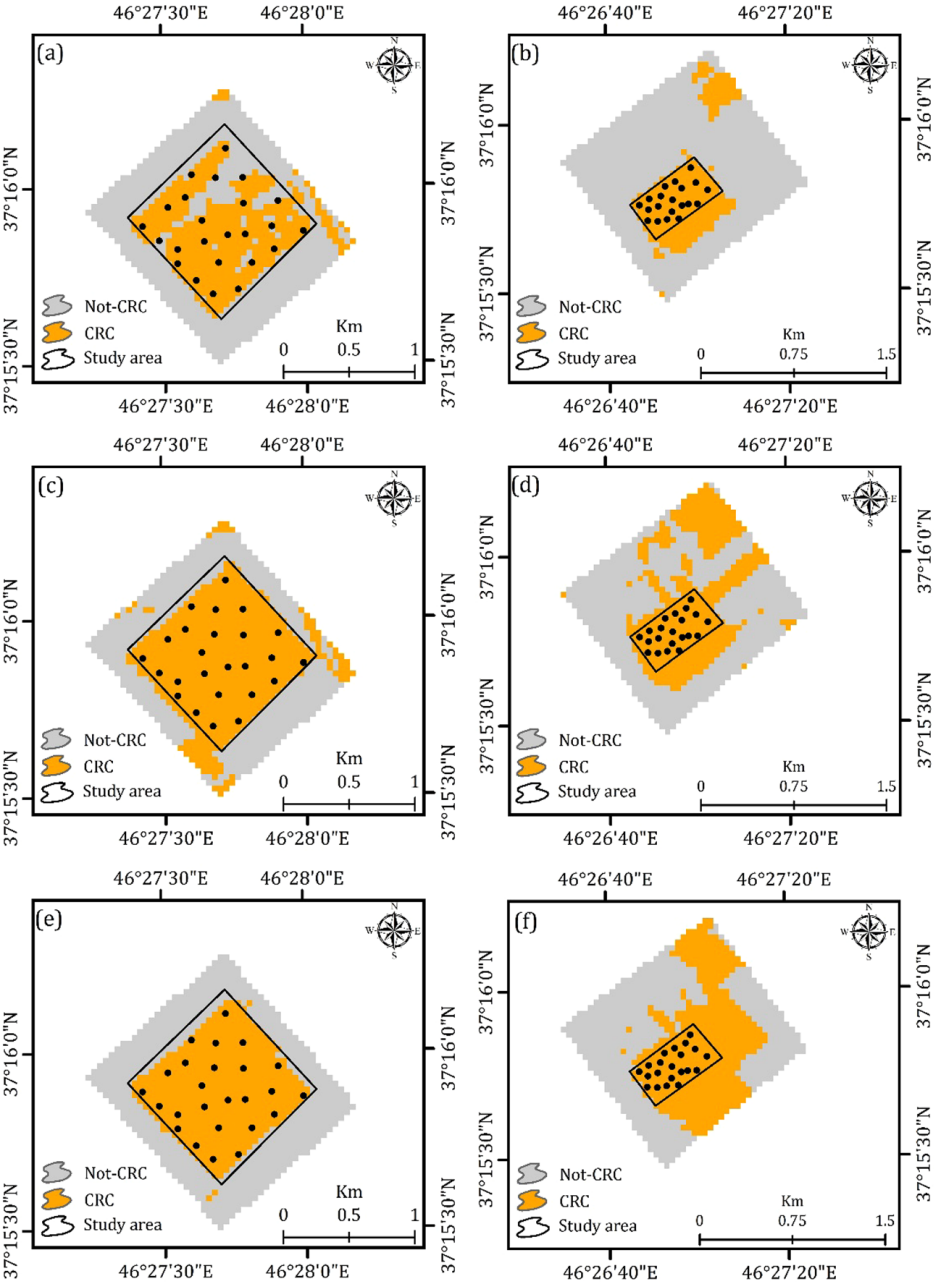
Generated CRC maps using an automated CNN approach in two classes in the ArcGIS 10.6 software (www. esri.com), namely not-CRC and CRC: (**a** and **b**) for the year 2013 for the case study areas 1 and 2, respectively, (**c** and **d**) for the year 2015 for the case study areas 1 and 2, respectively, and e and f) for the year 2021 for the case study areas 1 and 2, respectively.

This study applied an integrated approach of remote sensing and geospatial analysis for assessing the effects of CRC on agricultural productivity and soil chemical and physical characteristics. To this end, an automated CNN employed to detect CRC in two classes: not-CRC and CRC, for the years 2013, 2015, and 2021. A total of 275 GCPs were incorporated into the CRC models, with 70% for training and 30% for testing the results of CRC networks. The results show the high efficiency of CNN for detecting and mapping CRC. According to Table 3, CNN performed well in mapping CRC with an ACC of > 0.95 for the years 2013, 2015, and 2021. Figure 3 provides the results of applied CNN for detecting and monitoring CRC for the years 2013, 2015, and 2021.

**Figure 4:**
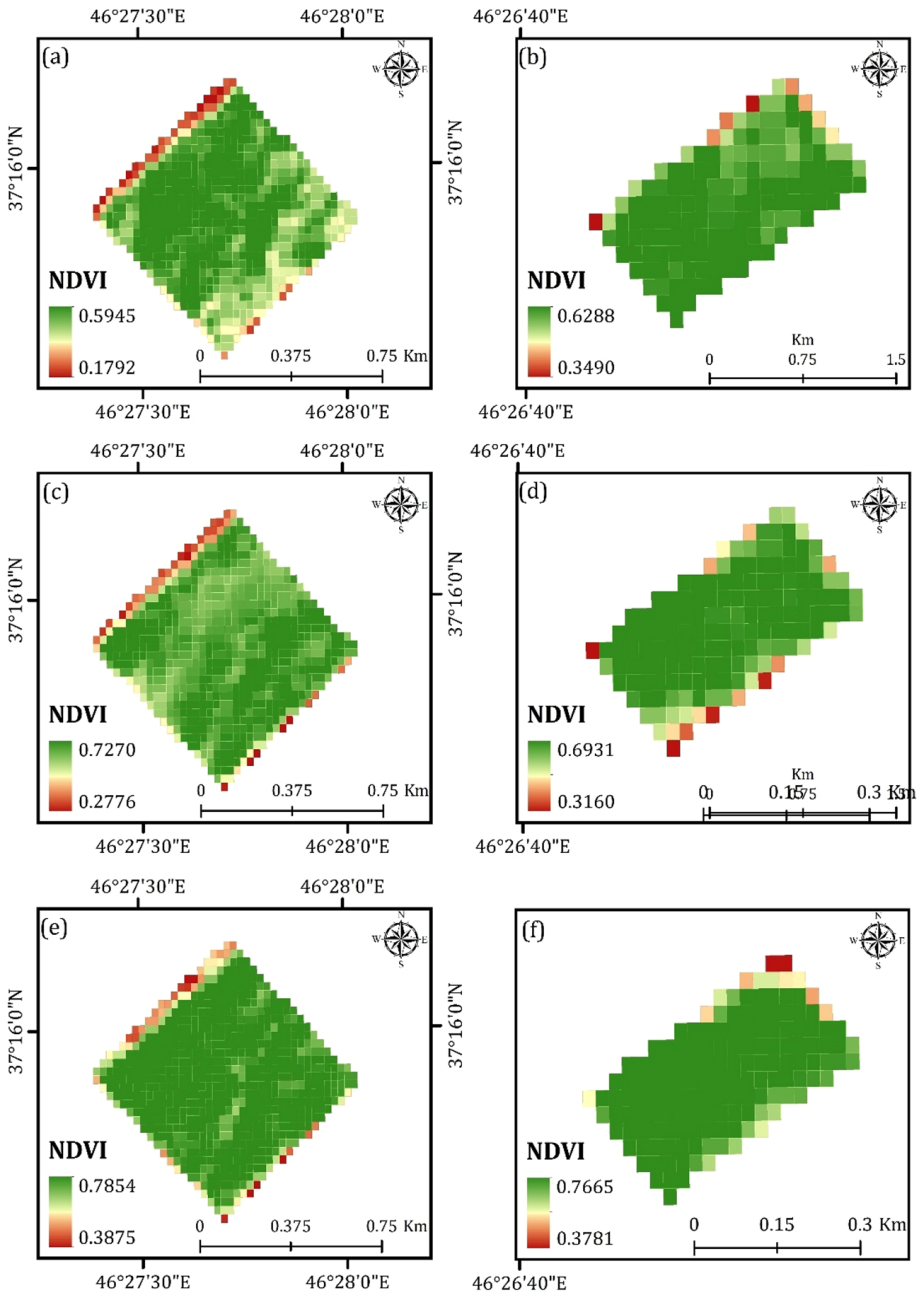
Generated vegetation cover maps using NDVI in the ArcGIS 10.6 software (www. esri.com): (**a** and **b**) for the year 2015 for the case study areas 1 and 2, respectively, (**c** and **d**) for the year 2019 for the case study areas 1 and 2, respectively, and (**e** and **f**) for the year 2022 for the case study areas 1 and 2, respectively.

A series of Landsat images was then used to evaluate vegetation status using NDVI for the years 2015, 2019, and 2022. Figure 4 reveals the results of NDVI in the study areas. This study also considered the effects of other predisposing variables, including precipitation, soil moisture, and soil temperature, on the changes in vegetation density for the years 2015, 2019, and 2022. The results reveal an increase in NDVI values from 2015 to 2022. According to Fig. 4, the highest NDVI values were 0.5945 and 0.6288 for the year 2015 in case study areas 1 and 2, respectively. However, the NDVI values increased by about 0.1909 and 0.1377 in case study areas 1 and 2, respectively for the year 2022 (as shown in Fig. 4). This means that the impacts of CRC on agricultural productivity may be positive. For being sure about the positive effects of CRC on agricultural productivity, monthly and annual changes in certain predisposing variables, including precipitation, soil moisture, and soil temperature, were evaluated. The results indicate no significant changes in precipitation, soil moisture, and soil temperature. As seen in Table 4, several points were created over the study areas, and the Zonal Statistics as Table tool in the Arc GIS 10.8 environment was used to assess the changes in precipitation, soil moisture, and soil temperature for the years 2015, 2019, and 2022. According to Table 4, the mean precipitation values were 331, 329, and 328 for study area 1 for the years 2015, 2019, and 2022. Similarly, the mean precipitation values for study area 2 were estimated at 252, 250, and 249 for the years 2015, 2019, and 2022 (Table 4). Regarding soil moisture, the mean values for study area 1 were 0.3804, 0.3919, and 0.3878 for the years 2015, 2019, and 2022, respectively. The mean soil moisture values were estimated at 0.4247, 0.4122, and 0.4214 for the same years, as shown in Table 4. Lastly, the mean soil temperature values in Kelvin (K) were 283.52 K, 282.61 K, and 283.21 K for study area 1 for the years 2015, 2019, and 2022, respectively. Similarly, for study area 2, the mean soil temperature values were 282.85 K, 281.86 K, and 281.98 K for the same years, as presented in Table 4.

**Table 4:** Annual precipitation (ml), soil moisture, and soil temperature (K) changes for the years 2015, 2019, and 2022.

Variable	Study area	2015	2019	2022
Precipitation	1	331	329	328
	2	252	250	249
Soil moisture	1	0.3804	0.3919	0.3878
	2	0.4247	0.4122	0.4214
Soil temperature	1	283.52	282.61	283.21
	2	282.85	281.86	281.98

## Discussion

### General discussion

CRC improves soil structure, increases organic matter content, enhances vegetation density, reduces evaporation, and aids in carbon dioxide fixation. Managing residues on agricultural land has a variety of positive effects on soil quality. Moreover, crop residues can be used for the production of biofuels^35,78^. Previous studies have not explored the effects of CRC on agricultural productivity and the chemical and physical characteristics of the soil. They have applied several spectral indexes, including LCA, SINDRI, NDI, and DFI, for detecting and mapping CRC. Moisture tends to darken soils and residue more equally in the visible bands, unless the residue is very fresh^79^. When attempting to use spectral differences (multi-band indices) for residue characterization, the influence of moisture on this process becomes more intricate. Under dry conditions, it is possible to distinguish the adsorption characteristics of crop residue from soil^80^. Well-calibrated broadband spectral indexes like Normalized Difference Tillage Index (NDTI) can accurately map CRC during such conditions. However, in wet conditions, differences between Landsat SWIR bands become less noticeable, causing these indexes to lose effectiveness. This is because moisture presence can both consistently lower reflectance across the SWIR spectrum and variably impact the reflectance (due to moisture absorption features) of both soil and residue, which may dry or wet at varying rates. Addressing both these challenges requires applying a moisture correction to NDTI to generate reliable percent residue assessments under high moisture conditions^81^. Recent studies have also applied learning-based approaches such as machine learning algorithms^32–34^ for detecting and mapping CRC. This study, on the other hand, employed an integrated approach of remote sensing and geospatial analysis to map and monitor the CRC and assess its impacts on agricultural productivity and soil chemical and physical characteristics. The findings highlight the high ability of automated CNN in detecting and mapping CRC. The outcomes of this study demonstrate the CNN's capacity for mapping CRC using Landsat remote sensing images. In comparison to traditional machine learning methods like support vector machines and neural networks, CNN mitigates the loss of edge information during the extraction of CRC from remote sensing images. Traditional machine learning techniques primarily rely on the accuracy of the extracted features, encompassing factors like pixel values, shapes, textures, and positions. Conversely, deep learning through convolutional neural networks can autonomously acquire pertinent high-level features directly from remote sensing images for CRC classification. This streamlines the process, lessening the need to design custom feature extractors for every classification task. The results of this study also reveal the positive impacts of CRC on agricultural productivity and soil fertility (See Fig. 4 and Tables 7 and 8).

### The impacts of CRC on vegetation density

As mentioned in previous sections, a primary objective of this study is to assess the effects of CRC on agricultural productivity. In this regard, the NDVI index applied to Landsat series images captured in the years 2015, 2019, and 2022 to map the greenness of the study areas. Subsequently, we established a total of 10 points for study area 1 and 8 points for study area 2. These points were strategically positioned within the ArcGIS 10.8 environment. In order to accurately quantify the effects of CRC on agricultural productivity, we utilized the Zonal Statistics as Table tool within the ArcGIS 10.8 environment. Tables 5 and 6 show the results of applied Zonal Statistics as Table tool over the study areas 1 and 2, respectively.

**Table 5:** Results of the Zonal Statistics as Table tool in the Arc GIS 10.8 environment for assessing the change in vegetation status for the years 2015, 2019, and 2022 for the study area 1.

Point	2015	2019	2022
1	0.5204	0.6089	0.7243
2	0.5090	0.5790	0.6601
3	0.5080	0.6186	0.7045
4	0.4069	0.6704	0.6980
5	0.4585	0.5972	0.6469
6	0.3562	0.5798	0.6173
7	0.5371	0.6987	0.7154
8	0.4436	0.5824	0.6334
9	0.3986	0.6368	0.6562
10	0.5285	0.6936	0.7250

**Table 6:** Results of the Zonal Statistics as Table tool in the Arc GIS 10.8 environment for assessing the change in vegetation status for the years 2015, 2019, and 2022 for the study area 2.

Point	2015	2019	2022
1	0.6149	0.6095	0.6704
2	0.6055	0.6305	0.6726
3	0.5961	0.5396	0.5904
4	0.6006	0.5951	0.7055
5	0.5951	0.6478	0.7543
6	0.6164	0.6518	0.7571
7	0.5888	0.5151	0.6749
8	0.6021	0.6164	0.7394

According to Table 5, for point 1, the NDVI mean value increased by about 0.2039 from 2015 to 2022 in study area 1. Similarly, the NDVI mean value for point 2 increased by about 0.1511 from 2015 to 2022, as shown in Table 5. As we can see in Table 5, for point 3, the NDVI mean value increased by about 0.1965 from 2015 to 2022. Point 4 also exhibited an increase in the NDVI mean value of about 0.2911 from 2015 to 2022, as indicated in Table 5. According to Table 5, for point 5, the NDVI mean value increased by about 0.1884 from 2015 to 2022. Point 6 depicted a similar trend with an increase of about 0.2611 in the NDVI mean value from 2015 to 2022. As we can see in Table 5, for point 7, the NDVI mean value increased by about 0.1783 from 2015 to 2022. The NDVI mean value also increased by about 0.1898 from 2015 to 2022 for point 8, as shown in Table 5. According to Table 5, for point 9, the NDVI mean value increased by about 0.2576 from 2015 to 2022. Similarly, the NDVI mean value for point 10 increased by about 0.1965 from 2015 to 2022, as shown in Table 5.

According to Table 6, for point 1, the NDVI mean value increased by about 0.0555 from 2015 to 2022 in study area 2. Similarly, the NDVI mean value for point 2 increased by about 0.0671 from 2015 to 2022, as shown in Table 6. As we can see in Table 6, for point 3, the NDVI mean value increased by about 0.0057 from 2015 to 2022. Point 4 also saw an increase in the NDVI mean value by about 0.1049 from 2015 to 2022, as indicated in Table 6. According to Table 6, for point 5, the NDVI mean value increased by about 0.1592 from 2015 to 2022 in study area 2. Point 6 exhibited a similar trend with an increase of about 0.1407 in the NDVI mean value from 2015 to 2022. As we can see in Table 6, for point 7, the NDVI mean value increased by about 0.0861 from 2015 to 2022. Lastly, point 8 indicated an increase in the NDVI mean value of about 0.1373 from 2015 to 2022, as shown in Table 6.

### The impacts of CRC on soil chemical and physical characteristics

Tables 7 and 8 show the results of CRC’s effects on soil chemical and physical characteristics. As we can see in Table 7, the EC level increased by about 0.04 and 0.02 from 2015 to 2022 in case study areas 1 and 2, respectively. According to Table 7, while the PH decreased by about 0.02 for study area 1 from 2015 to 2022, it increased by about 0.03 for study area 2. The Na values also exhibited an increase of about 0.02 and 0.01 for study areas 1 and 2, respectively, during the same period, as shown in Table 7. As we can see in Table 7, the Mg level increased by about 0.08 and 0.06 from 2015 to 2022 in case study areas 1 and 2, respectively. The HCO_3_ values also shown an increase of about 0.07 and 0.22 for study areas 1 and 2, respectively, from 2015–2022, as shown in Table 7. According to Table 7, while the K level decreased by about 0.01 for study area 1 from 2015 to 2022, it increased by about 0.01 for study area 2.

**Table 7:** Results of the collected GCPs from the study areas for assessing the effects of CRC on soil chemical characteristics from 2015 to 2022.

Soil properties	Case study area 1			Case study area 2		
	2015	2019	2022	2015	2019	2022
EC	0.59	0.61	0.63	0.49	0.50	0.51
PH	7.11	7.09	7.09	6.78	6.80	6.81
Na	0.56	0.57	0.58	0.53	0.53	0.54
Mg	1.68	1.75	1.76	1.33	1.35	1.39
HCO_3_	3.73	3.78	3.80	2.35	2.58	2.57
K	0.13	0.12	0.12	0.11	0.12	0.12

**Table 8:** Results of the collected GCPs from the study areas for assessing the effects of CRC on soil physical characteristics from 2015 to 2022.

Soil properties	Case study area 1			Case study area 2		
	2015	2019	2022	2015	2019	2022
Sand	25.14	25.19	25.31	30.48	30.89	31.02
Silt	32.85	32.99	33.01	67.25	67.87	68.12
Clay	8.44	8.64	8.87	7.89	7.90	7.90

As seen in Table 8, the amount of sand increased by about 0.21 and 0.54 in case study areas 1 and 2, respectively, from 2015 to 2022. Similarly, the amount of silt also increased by about 0.16 and 0.87 in the study areas 1 and 2, respectively during the same period, as shown in Table 8. According to Table 8, there was an increase of 0.43 and 0.01 in clay content for study areas 1 and 2, respectively, between 2015 and 2022.

### Limitation of this study

This study developed an integrated approach of remote sensing and geospatial analysis for monitoring and mapping the effects of CRC on agricultural productivity and soil chemical and physical characteristics. Despite the satisfying results obtained from this work, there exist two primary limitations that future research in the domain of CRC and its impacts on vegetation density and soil fertility could address. Firstly, the current study relied on Landsat series images with a spatial resolution of 30 m. In future endeavors, the availability of Sentinel-2 images could be exploited as a potential alternative for a more extended time period, aiding in the detection and mapping of CRC. Secondly, a recommended avenue for forthcoming research is the adoption of an integrated methodology that combines object-based image analysis and Convolutional Neural Networks (CNNs) to monitor CRC changes. This amalgamation is noteworthy because object-based image analysis harnesses numerous features, such as brightness, to detect specific features. This comprehensive approach enhances the precision and accuracy of the outcomes obtained. In conclusion, this study's innovative approach has provided valuable insights into the effects of CRC on agricultural systems. The outlined limitations offer a roadmap for subsequent research endeavors to build upon and further refine our understanding of CRC’s impact on vegetation density and soil fertility.

## Conclusion

This study introduced an integrated approach combining remote sensing and geospatial analysis to effectively identify and map CRC while also evaluating its impact on vegetation density, as well as soil chemical and physical attributes. The results exhibit the remarkable efficiency of an automated CNN data-driven approach (> 0.96) in detecting and mapping CRC. Furthermore, the outcomes underscore the positive influence of CRC on vegetation density and soil chemical and physical characteristics.

The findings of this study demonstrate that the integration of remote sensing and geospatial analysis is adept at accurately detecting and mapping CRC while simultaneously assessing its effects on vegetation status and soil fertility. The efficiency of Landsat series images in mapping and monitoring dynamic features like vegetation and soil is also emphasized in this research.

The study underscores the efficacy of learning-based methods in proficiently classifying Earth’s features. When paired with satellite-based datasets and Geographic Information Systems (GIS), this computer-based approach proves to be notably efficient. Importantly, the research reveals that crop residue cover contributes to heightened soil productivity in terms of chemical and physical attributes. Moreover, it serves as means to maintain soil moisture levels, a critical factor for crop survival, particularly in semi-arid and arid regions.

The insights from this study hold significant value for researchers engaged in soil science and agriculture. Additionally, the findings provide essential information for decision-makers and planners involved in land management and soil erosion control. Ultimately, our research contributes to enhancing the understanding of the multifaceted impacts of CRC, benefiting both scientific advancements and practical land management strategies.

## Data Availability

The datasets used and/or analysed during the current study available from the corresponding author on reasonable request.
